# Ferromagnetism in Two-Dimensional Dysprosium–Platinum Surface Alloy

**DOI:** 10.1021/acs.nanolett.5c00262

**Published:** 2025-05-17

**Authors:** Marta Przychodnia, Maciej Bazarnik

**Affiliations:** † Institute of Physics, Poznan University of Technology, 3 Piotrowo Street, 60-965 Poznan, Poland; ‡ Institute of Physics, Münster University, Wilhelm-Klemm-Str. 10, 48149 Münster, Germany

**Keywords:** surface alloys, 2D ferromagnetism, STM/STS, moiré, rare earth metals

## Abstract

In this study, we comprehensively analyze single and triple layers of a new two-dimensional surface alloy, namely DyPt_2_. Both are ferromagnetic materials with an in-plane easy magnetization axis and low Curie temperature on the order of a few Kelvins. Magnetic and electronic properties confirm weak interlayer coupling and the dominance of interactions within alloy layers. Atomic-scale investigation proved nearly the same atomic structure of the termination layer and varying moiré patterns. The electronic structures of single and triple layer DyPt_2_ are similar, consisting of a mixture of Dy and Pt electronic states. The intensity of these electronic states varies within the moiré pattern, similar to the surface local work function, demonstrating modulated coupling between the surface alloy and the substrate. The presented results provide essential knowledge for further research of this system in terms of its application in the growth of densely packed arrays of magnetic clusters and molecules.

## Graphical abstract

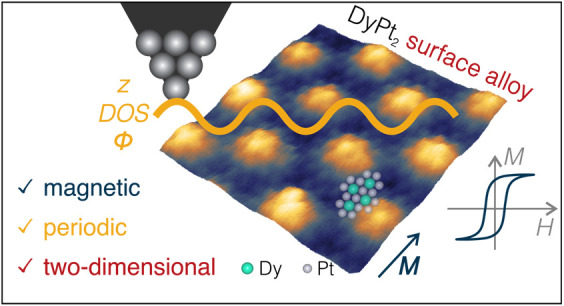

Surface alloys of REM (rare earth metal) with NM (noble metal) make up a wide group of 2D, magnetic, and structurally templated materials with peculiar properties arising from their size confinement. For example, in the bulk GdAu_2_, frustration leads to antiferromagnetic interlayer coupling, while the surface alloy is ferromagnetic.^1^ A well-known group of REM-Au, -Ag, and -Cu surface alloys share the same structure of REM–NM_2_ with coexisting short-range atomic order and long-range order (moiré pattern).^1–4^ As reported for the GdAu_2_ surface alloy, such a structure has been successfully implemented for the templated growth of densely packed magnetic arrays.^5–7^ Moreover, due to the REMs, known for their high magnetic moments, these materials also exhibit magnetic properties dependent on the elements involved in surface alloy formation.^8–10^ Not only REM but also the substrate affect the magnetic properties of the surface alloy. For Gd–NM systems, it has been proven that changing the substrate from Au to Ag causes a significant increase of Curie temperature (*T*_C_) from 19 to 85 K.^8^ Pt is another NM that is easy to magnetically polarize,^11^ and when used as a substrate, can enhance the magnetic interactions between REM atoms.^12^ Compared to the other NMs, surface alloys involving Pt are less known and the literature on experimental research is limited to Ce–Pt,^13–17^ La–Pt,^18^ and Gd–Pt^12^ systems. REM-Pt surface alloys reveal a wider structural variety forming most commonly REM–Pt_5_,^12,15,16,19^ REM–Pt_6_,^17^ and REM–Pt_2_,^12,14^ but also more complex multilayered structures.^14,20^ All of these surface alloys exhibit short- and long-range orders; therefore, they may support templated growth of densely packed magnetic arrays similar to GdAu_2_ and GdAg_2_ surface alloys. In this investigation, we employed Dy as a REM in surface alloy due to its high magnetic moment of 10.65 μ_B_.^21,22^ At the same time, it has relatively high *T*_C_ = 85–89 K^21,22^ and in the ferromagnetic state Dy shows an axial anisotropy with magnetic moments confined to the basal plane.^22^ The following report focuses on the structure, electronic properties, and, in particular, magnetic properties, of the DyPt_2_ surface alloy. We investigate how the moiré pattern influences electronic states and the local work function. Additionally, we analyze the magnetic order as well as the spin and orbital magnetic moments, along with the Curie temperature of two thicknesses of the DyPt_2_ surface alloy. Our analysis emphasizes the potential of this surface alloy as a future substrate for studying magnetic molecules and for the templated growth of densely packed magnetic cluster arrays.

The previous study^12^ showed that REM-Pt(111) surface alloys grow the best when a reactive growth is employed. In this scheme, formation of Dy–Pt surface alloy is observed between 780 ± 5 and 1165 ± 5 K. Below this range, Dy on Pt(111) forms Dy–Pt clusters and islands with unknown stoichiometry, as observed for the Gd–Pt system.^12^ Above 1165 K the surface alloy is overheated and becomes disturbed. A similar behavior is observed for GdPt_*x*_ alloy above 773 K (500 °C).^20^ The effect, as explained by Ulrikkeholm et al.,^20^ is assigned to the interdiffusion of REM atoms and formation of Pt overlayers.

Identification of observed compounds is based on the comparison of their structure, electronic properties, growth parameters (Dy coverage and substrate temperature), and share of a given compound in the sample area occupation. On that basis, we find a single layer DyPt_2_ (1 L DyPt_2_) and a triple layer DyPt_2_ (3 L DyPt_2_), as shown in Figure 1. Both thicknesses of Dy–Pt surface alloy adopt the structure with the formula REM-NM_2_ similarly to the already well-known group of surface alloys involving Au,^1,9,10,23,24^ Ag,^8,25^ Cu,^4,25^ and Pt,^12,14^ as a substrate. Deposition of 0.4 monolayer (ML) Dy results in the dominance of 1 L DyPt_2_. Increased Dy coverage leads to the formation of multilayer islands pointing to the Stranski–Krastanov growth mode. Preparation of the sample dominated by 3 L DyPt_2_ requires therefore high loadings of Dy at the level of 2.7 MLs. Detailed analysis of sample area occupancy by Dy–Pt compounds depending on the growth conditions is described in Supplementary Note 1. Under certain conditions, it is possible to prepare a sample mostly covered by the chosen thickness of the surface alloy. Temperature-dependent studies of the surface morphology with nearly 0.4 ML coverage do not reveal Pt kagomé overlayer formation and transformation of 1 L DyPt_2_ into 1 L DyPt_5_ (see Supplementary Note 2). This shows the unprecedented thermal stability of DyPt_2_ compared to those of other REM-Pt surface alloys.

**Figure 1:**
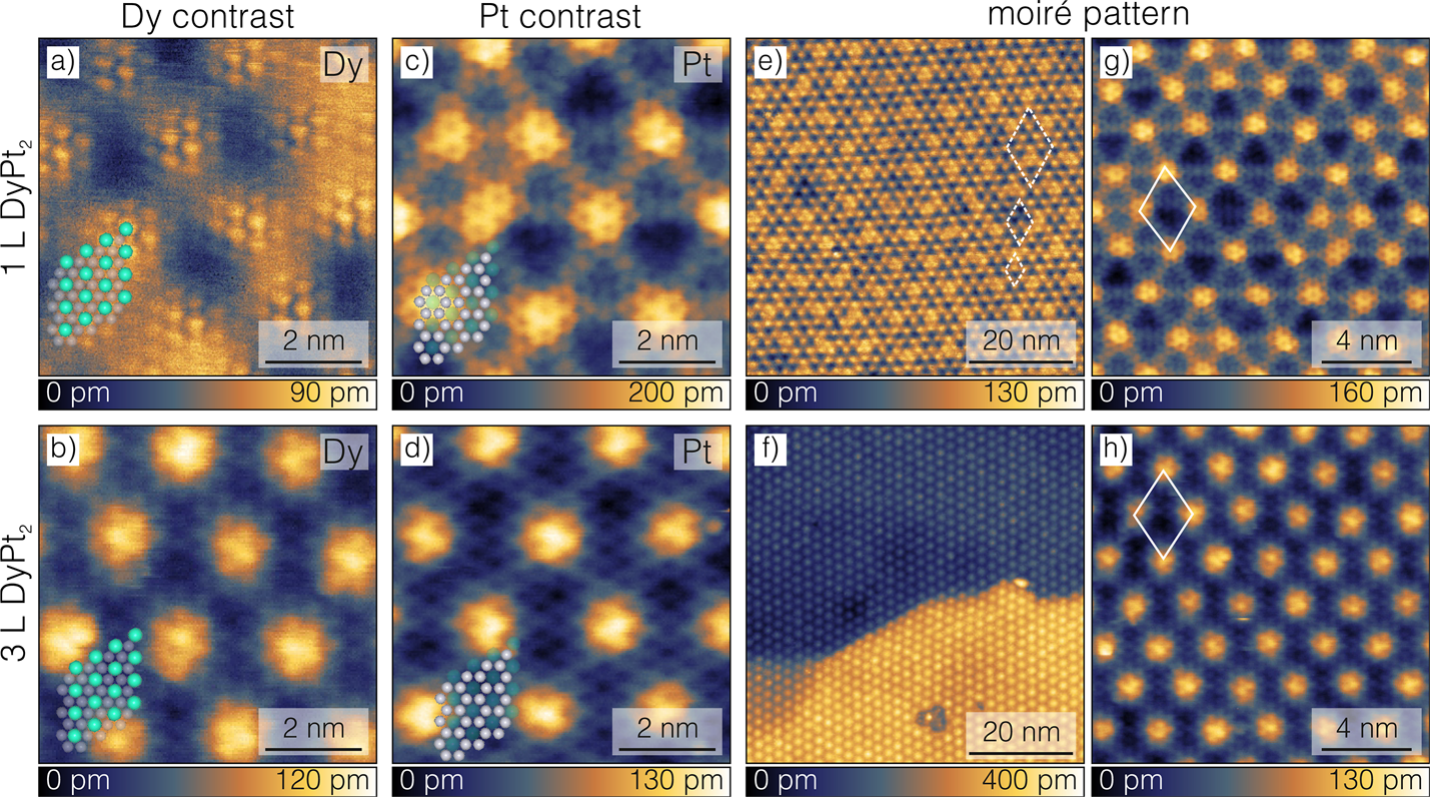
STM topography images of 1 L DyPt_2_ and 3 L DyPt_2_ surface alloy. Top and bottom rows present 1 L DyPt_2_ and 3 L DyPt_2_, respectively. (a–d) Atomic resolution topographies exposing separately Dy (a and b) as well as Pt (c and d) contrasts. The termination layer model is overlaid on the topographies, with turquoise and gray spheres presenting Dy and Pt atoms, respectively. (e and f) Large-scale STM topographies of moiré patterns. White dashed rhombus in (e) indicates the three most common periodicities of the moiré unit cell. (g and h) STM topographies of moiré unit pattern and atomic resolution contrast. White rhombus indicates reference moiré unit cells. Tunneling parameters: (a) *I*_t_ = 1.5 nA, *U* = 2 V, (b) *I*_t_ = 10 nA, *U* = 3 V, (c) *I*_t_ = 5 nA, *U* = 2 mV, (d) *I*_t_ = 5 nA, *U* = 3 V, (e) *I*_t_ = 1 nA, *U* = 2 V, (f) *I*_t_ = 0.5 nA, *U* = – 2 V, (g) *I*_t_ = 5 nA, *U* = 10 mV, (h) *I*_t_ = 10 nA, *U* = 3 V.

Atomic resolution scanning tunneling microscopy (STM) images reveal two contrasts separately exposing Dy (Figure 1a,b) and Pt atoms (Figure 1c,d). Dy atoms form a hexagonal lattice with the nearest neighbor distance determining the size of a unit cell yielding $a_{1{LDyPt}_{2}}=510\pm 2$ pm for 1 L DyPt_2_ and $a_{3{LDyPt}_{2}}=515\pm 2$ pm for 3 L DyPt_2_. Obtained values are comparable with the lattice constant of bulk polycrystalline DyPt_5_ alloy, which is 523.8 pm.^26^ Bearing in mind the structures of DyPt_5_ and DyPt_2_, the lattice constants of both should share similar dimensions. The lattice constants of DyAu_2_ and DyAg_2_ in polycrystalline form (522.4 and 522.7 pm, respectively^27^) and in surface alloy form (550 and 520 pm, respectively^25^) are also in line with our observation. Pt atoms surrounding Dy atoms form a kagomé lattice with Dy atoms arranged in kagomé’s holes. Both 1 L DyPt_2_ and 3 L DyPt_2_ are terminated with an intermixed DyPt_2_ layer of similar dimensions, making the distinction between them on an atomic scale challenging. The difference between both compounds is more pronounced in large-scale STM topography images exposing their moiré patterns (see Figure 1e–h). The one observed for 1 L DyPt_2_ (Figure 1e) closely resembles a nonuniform moiré pattern of 1 L GdPt_2_ ^12^ exposing aperiodic lines and defects beneath the surface of the alloy. The moiré pattern of 3 L DyPt_2_ (Figure 1f) shows a higher degree of order, without aperiodic lines, and lower visibility of subsurface defects. Ordering of the moiré pattern together with an increase of the atomic lattice constant with increasing alloy layer numbers points to the relaxation of the structure. A closer look at the STM images showing both the atomic structure and the moiré pattern reveals differences in atomic order relative to the moiré pattern pointing out that structures are incommensurate (see the top sites of moiré patterns in Figure 1g,h). Moiré unit cells marked with white solid line rhombuses are therefore only a reference for further analysis and are not actual moiré unit cells. Their dimensions are comparable within measurement accuracy (2.2 ± 0.2 nm for 1 L DyPt_2_ and 2.1 ± 0.2 nm for 3 L DyPt_2_). In Figure 1e, one can distinguish three main periodicities. They correspond to contracted (2 × 2), relaxed (2 × 2), and (4 × 4) periodicities of reference moiré pattern’s unit cells, also observed in a low-energy electron diffraction (LEED) experiment (see Supplementary Note 3). Upon Dy deposition, there are no areas of bare platinum left. Therefore, based on STM images of surface alloy only, it is not possible to directly determine the rotation angle between the alloy and the substrate. The LEED experiment, however, gave us insight into this relation by exposing two rotational domains: parallel and rotated by 30 ± 3° relative to the substrate. Rotational domains were already observed for Ce–Pt thin film alloys^14,15,28,29^ as well as Gd–Pt surface structures.^20^ The coexistence of rotational domains indicates that both configurations are energetically equivalent or similar.

We investigate the influence of the moiré pattern on electronic properties by recording 32 constant-height (CH) scanning tunneling spectra (STS) evenly spaced along the line crossing through the moiré pattern (Figure 2a). Results for both thicknesses of surface alloy reveal a similar electronic structure and moiré dependence, which is expected due to the same composition of the termination layer. In Figure 2a) we present results for 1 L DyPt_2_ as a waterfall plot (for 3 L DyPt_2_ results see Supplementary Note 4). The *x*-axis presents the bias *U* and the *y*-axis lateral position, and the normalized d*I*_t_/d*U* signal intensity is color-coded. The most pronounced variation observed across the moiré unit cell is the d*I*_t_/d*U* signal intensity modulation. We observe minor energetic position shifts of electronic states. The maximum difference between extremely shifted states is Δ_u_ = 0.18 V for the unoccupied side, and it is smaller than for the occupied side where Δ_o_ = 0.34 V. Based on the detailed analysis of the energetic shifts of electronic states (see Supplementary Note 5), two curves with extreme energetic positions of the electronic state observed around 2.75 V are selected and separately presented in Figure 2b). The peak shifted toward lower energy corresponds to the top site of the moiré unit cell, while the peak shifted toward higher energy is assigned to the bottom site. Within the ±3 V range one pronounced maximum of occupied electronic states and two main maxima of unoccupied electronic states are observed. We attribute the peak at around −2.55 ± 0.05 V to Pt d states.^30^ Compared to the similar system of 1 L GdPt_2_ the electronic states observed below the Fermi level are assigned to the combination of 5d and 6s states of Pt, with the domination of the former one.^12^ The most pronounced signal observed for the unoccupied electronic states combines 2.55 ± 0.05 and 2.85 ± 0.05 V peaks with the dominance of the former. Although for these energies one could expect REM 4f states, they are not observed in STS due to their sharp dropoff to the vacuum. We attribute these states mostly to 5d states of REM.^12^ 1 L DyPt_2_ has a single peak with relatively high intensity at 1.45 ± 0.05 V and two smaller peaks at 0.60 ± 0.05 and 0.85 ± 0.05 V. The lower intensity peaks are the combination of 5d and 6s states of REM and Pt.^12^

**Figure 2:**
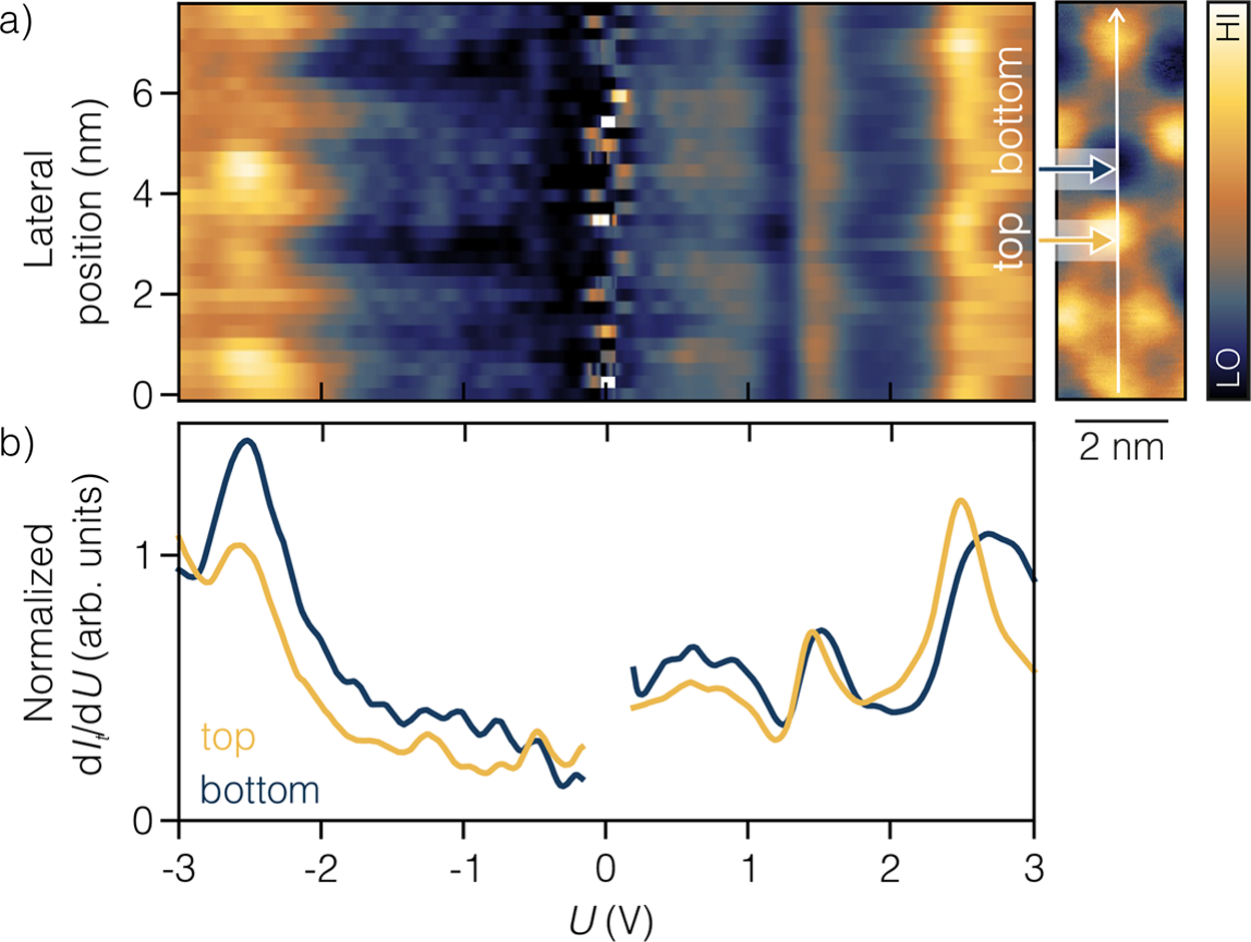
Moiré pattern influence on electronic properties of 1 L DyPt_2_. (a) Waterfall plot of 32 normalized CH STS spectra taken along the white arrow marked in STM topography on the right side. The noise around 0 V bias results from the normalization procedure. (b) Two d*I*_t_/d*U* curves selected from the data set shown in (a) for the two extreme positions of the peak around 2.75 V corresponding to the top (yellow) and the bottom (blue) positions on the moiré pattern. The spectra positions are also marked in the topography with arrows. The energy range around 0 V bias was omitted due to the normalization procedure noise. Tunneling parameters: *I*_stab_ = 5 nA, *U*_stab_ = 3 V, *U*_mod_ = 50 mV, ω_mod_ = 49.95 kHz.

Constant-current (CC) STS taken in the energy range between 1 and 10 V, shown in Figure 3a, exposes five image potential states (IPSs) with dependence on position within the moiré pattern. The peaks observed below 5 V, as described above, correspond mostly to the 5d states of Pt. Figure 3b summarizes work function values obtained within the moiré pattern at 32 evenly spaced points. The spectra corresponding to the two most extreme values of the work function ( $\phi_{1{LDyPt}_{2}-\min}$ = 3.78 ± 0.05 eV and $\phi_{1{LDyPt}_{2}-\max}$ = 4.12 ± 0.05 eV) are presented in Figure 3c with blue and yellow colors accordingly. Figure 3d shows corresponding plots of the IPS positions as a function of their order ${\left(n-\frac{1}{4}\right)}^{2/3}$ , used to extract the values. The inset in Figure 3d shows a zoom-in to the *y*-intercept of the linear fitting to indicate the values of the local work function. Similarly to other surface alloys (TbAu_2_, HoAu_2_, ReAu_2_,^31^ 1 L GdAu_2_,^32^ and 2 L GdAu_2_ ^1^), the work function of NM is reduced (pure Pt(111) is *ϕ*_Pt(111)_ = 5.77 eV^33^). As the substrate has a higher work function than the surface alloy, we assume that the higher value of $\phi_{1{LDyPt}_{2}}$ accounts for the regions of the moiré pattern with stronger hybridization to the substrate pointing to the strongest coupling of bottom sites and the weakest coupling of top sites.

**Figure 3:**
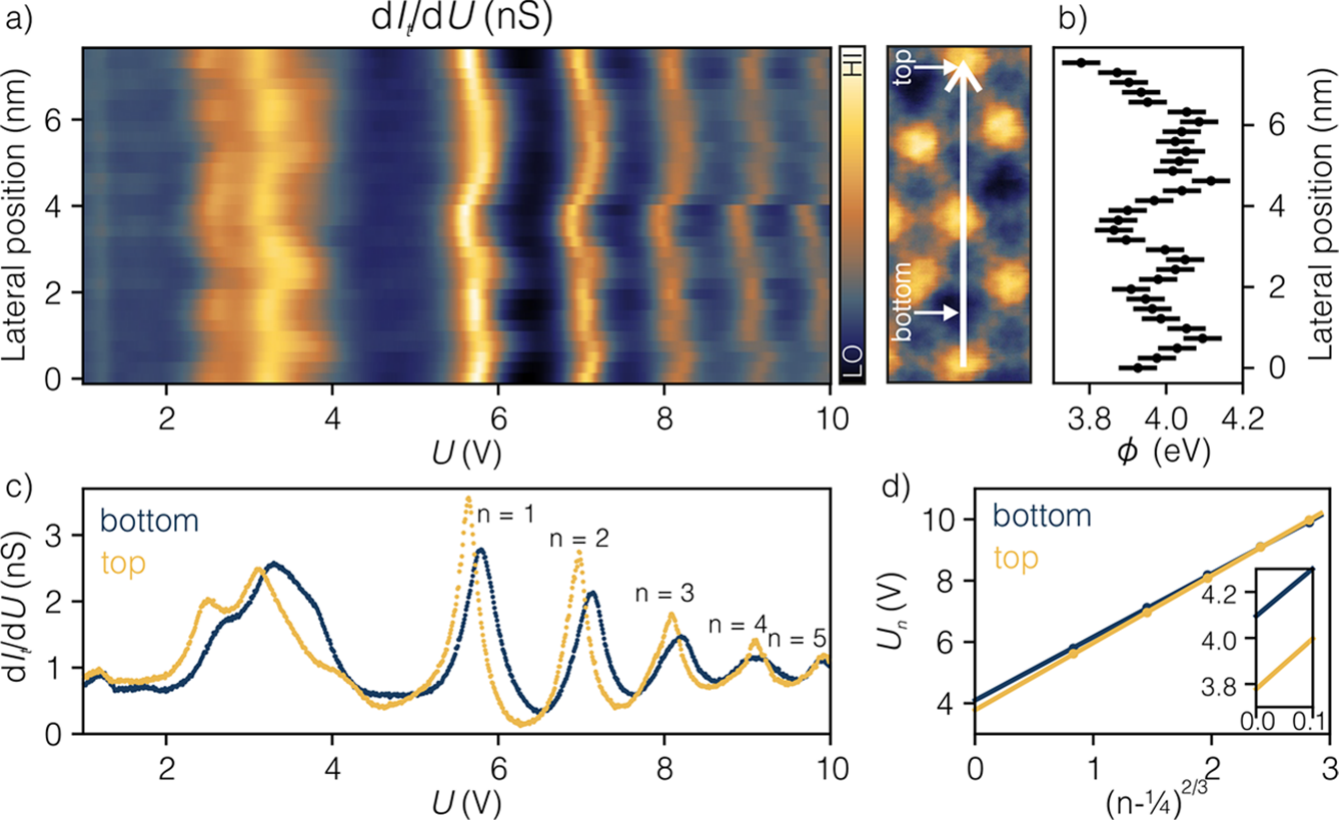
CC d*I*_t_/d*U* spectra of 1 L DyPt_2_. (a) Waterfall plot of 32 CC STS for bias in the range from 1 to 10 V taken along the moiré unit cell. The trace is marked with a white arrow in the STM topography. Tunneling parameters: *I*_stab_ = 1 nA, *U*_stab_ = 4 V, *U*_mod_ = 50 mV, ω_mod_ = 6777 Hz. (b) Plot of the surface work function values across the moiré unit cell. (c) d*I*_t_/d*U* curves selected from the data set shown in (a) for the two extreme work function values, that correspond to the top (yellow) and bottom (blue) positions of the moiré pattern. (d) Plot of the IPS positions *U*_n_ as a function of IPS quantum number *n*. Points, the energy of the IPSs states extracted from (a); lines, linear fitting.

Magnetic properties of 1 L DyPt_2_ and 3 L DyPt_2_ are studied by X-ray magnetic circular dichroism (XMCD) at the Dy M_4,5_ absorption edges. Insets in Figure 4a,b show X-ray absorption spectra (XAS) recorded using the left (red line) and right (blue line) circularly polarized light. Presented XAS data are taken for in-plane geometry with an external magnetic field of μ_0_ *H* = 6.8 T applied along the beam direction. Out-of-plane geometry XAS is described in Supplementary Note 6. The difference between positive and negative XAS (the XMCD signal) for in-plane (yellow) and out-of-plane (blue) geometries is presented in Figure 4a,b for 1 L DyPt_2_ and 3 L DyPt_2_, respectively. The dichroism is observed for both beam–sample configurations and both thicknesses of surface alloy. Within the investigated energy range, two pronounced absorption channels, corresponding to M_5_ and M_4_ edges, are observed, confirming d state splitting. A stronger dichroism is observed for the M_5_ edge. The M_5_ edge is split into two extrema: a minimum at 1284.9 ± 0.1 eV and a maximum at 1288.9 ± 0.1 eV, while the M_4_ edge has only a maximum at 1322.1 ± 0.1 eV. The photon energies corresponding to the absorption edges are the same within the energy accuracy for both thicknesses of surface alloy, regardless of the beam–sample configuration, confirming the same chemical environment for both surface alloys. The signal intensity of the in-plane geometry XMCD is significantly higher than for the out-of-plane geometry for both thicknesses of the surface alloy due to a nonzero orbital magnetic moment and intrinsic anisotropy of Dy.^22,34^

**Figure 4:**
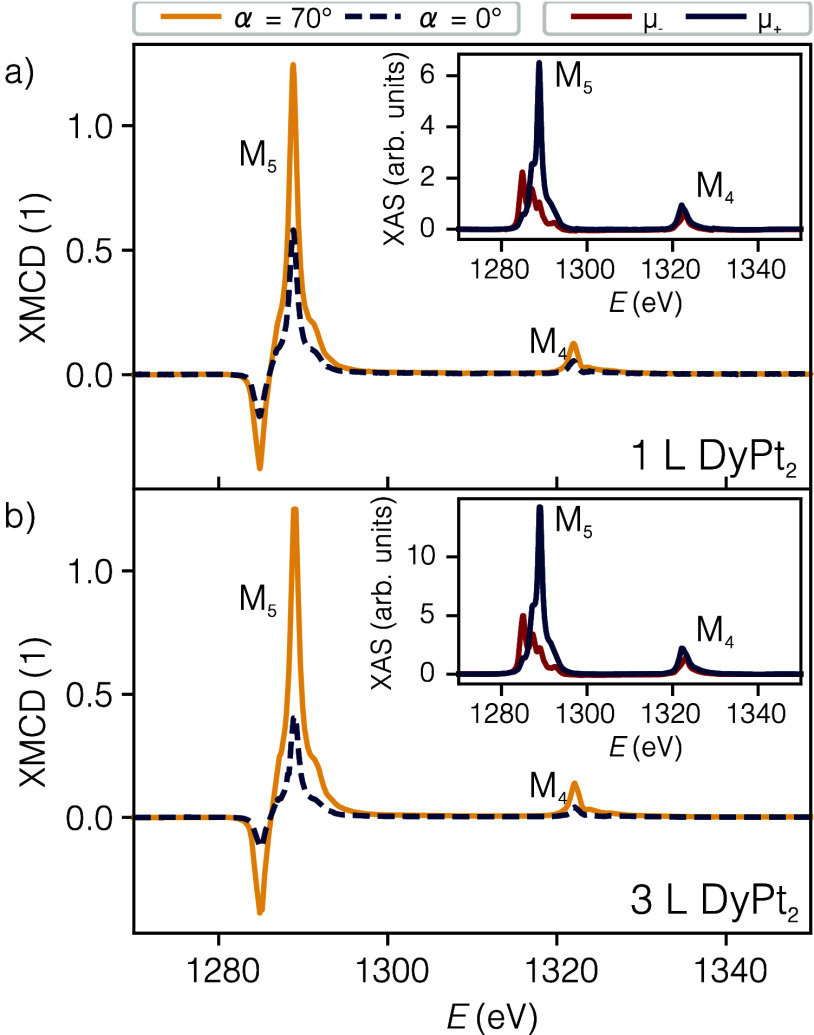
XAS/XMCD experiment results taken at 3 K and 6.8 T for (a) 1 L DyPt_2_ and (b) 3 L DyPt_2_. XMCD spectra for normal (blue) and grazing (yellow) light incidence configurations in the energy range from 1270 to 1350 eV. Insets: grazing geometry XAS spectra for positive (blue) and negative (red) light polarization.

Using magneto-optical sum rules for M_4,5_ edges^35–38^ both spin and orbital magnetic moments are calculated. Table 1 summarizes orbital, spin, and total magnetic moments presented in refs 21, 22, 34, and 39 together with the results extracted from our studies. The magnetic dipole operator for Dy is assumed as 0.15 ^39^ and the number of the holes as 5. The orbital magnetic moments are $m_{l-1{LDyPt}_{2}}=3.80\pm 0.01$ μ_B_ and $m_{l-3{LDyPt}_{2}}=3.81\pm 0.01$ μ_B_. The value is substantial, as expected for Dy, but it is significantly lower than simulated for the Dy^3+^ ion (5.11 μ_B_ ^39^) and observed experimentally for Dy atoms adsorbed on Pt(111) (4.1 ± 0.2 μ_B_ ^34^). The spin magnetic moments are $m_{s-1{LDyPt}_{2}}=3.32\pm 0.01$ μ_B_ and $m_{s-3{LDyPt}_{2}}=3.28\pm 0.01$ μ_B_. The values are again lower than simulated for Dy^3+^ ion (−4.48 μ_B_ ^39^) but higher than values obtained experimentally for Dy atoms adsorbed on Pt(111) (2.7 ± 0.1 μ_B_ ^34^). Finally, the total magnetic moments extracted from our experiment are lower ( $m_{1{LDyPt}_{2}}=7.12\pm 0.01$ μ_B_ and $m_{3{LDyPt}_{2}}=7.09\pm 0.01$ μ_B_) than the literature value of Dy^3+^ ion (10.65 μ_B_ ^21,22^ ) and higher than for Dy atoms adsorbed on Pt(111) (6.8 ± 0.2 μ_B_ ^34^). Lowering of the total magnetic moment may be caused by charge transfer to the Pt atoms of alloy and substrate layers, as it was already observed for a similar Gd–Pt system, where the polarization reached down to the second layer of the substrate.^12^

**Table 1:** Summary Table of Orbital (*m*_l_), Spin (*m*_s_), and Total (*m*) Magnetic Moments Obtained in Refs 21, 22, 34, and 39 Together with the Results of the Study Presented Here

	*m*_l_ (μ_B_)	*m*_s_ (μ_B_)	*m* (μ_B_)
1 L DyPt_2_	3.80 ± 0.01	3.32 ± 0.01	7.12 ± 0.01
3 L DyPt_2_	3.81 ± 0.01	3.28 ± 0.01	7.09 ± 0.01
Dy atoms on Pt(111)	4.1 ± 0.2 ^34^	2.7 ± 0.1 ^34^	6.8 ± 0.2 ^34^
Dy^3+^	5.11 ^39^	4.48 ^39^	10.65 ^21,22^

Figure 5a,b shows magnetization curves after normalization taken at the M_5_ edge of 1 L DyPt_2_ and 3 L DyPt_2_, respectively, for normal (blue) and grazing (yellow) incidence angles. The shape of the curves points that both thicknesses of surface alloy are ferromagnetic with an in-plane easy magnetization axis. The in-plane arrangement of 1 L DyPt_2_ points to the strong influence of intrinsic anisotropy of Dy on the magnetic order of the surface alloy. Additionally, the same easy magnetization axis of 1 L DyPt_2_ and 3 L DyPt_2_ exposes the influence of the crystal field, pointing out that the interaction between Dy atoms within the same plane is stronger than the interlayer coupling. This could also point out that contrary to the bulk DyPy_2_ structure,^40^ adjacent layers are shifted with respect to each other, so the Dy atoms are not placed directly one above another. The magnetization signal saturates at 0.45 and 0.3 T for grazing incidence geometry for 1 L DyPt_2_ and 3 L DyPt_2_, respectively (see the insets of Figure 5a,b). Although it saturates below 1 T, wide magnetic field range hysteresis loops revealed that the signal still linearly increases with increasing magnetic field. As will be discussed later, the measurement temperature of 3 K is close to the *T*_C_ of both thicknesses of the surface alloy, and therefore the curves show a slight paramagnetic character. The coercive field is $H_{C-1{LDyPt}_{2}}=30\pm 2$ mT and $H_{C-3{LDyPt}_{2}}=20\pm 2$ mT for 1 L DyPt_2_ and 3 L DyPt_2_, respectively, indicating that 1 L DyPt_2_ is a harder ferromagnet than 3 L DyPt_2_. The values are significantly lower than the coercive field of the bulk DyPt_2_ alloy (66 mT^41^) and higher than local *H*_C_ obtained using spin-polarized STM for GdAu_2_ (17.5 mT^9^). For bulk Dy, between *T*_C_ and *T*_N_, magnetic moments are aligned ferromagnetically within individual planes, while they are rotated by a certain, temperature-dependent angle for successive planes forming a spiral structure.^21,22^ Here, the reduction of *H*_C_ most likely has a similar origin, where the interlayer coupling in 3 L DyPt_2_ reduces the coercive field.

**Figure 5:**
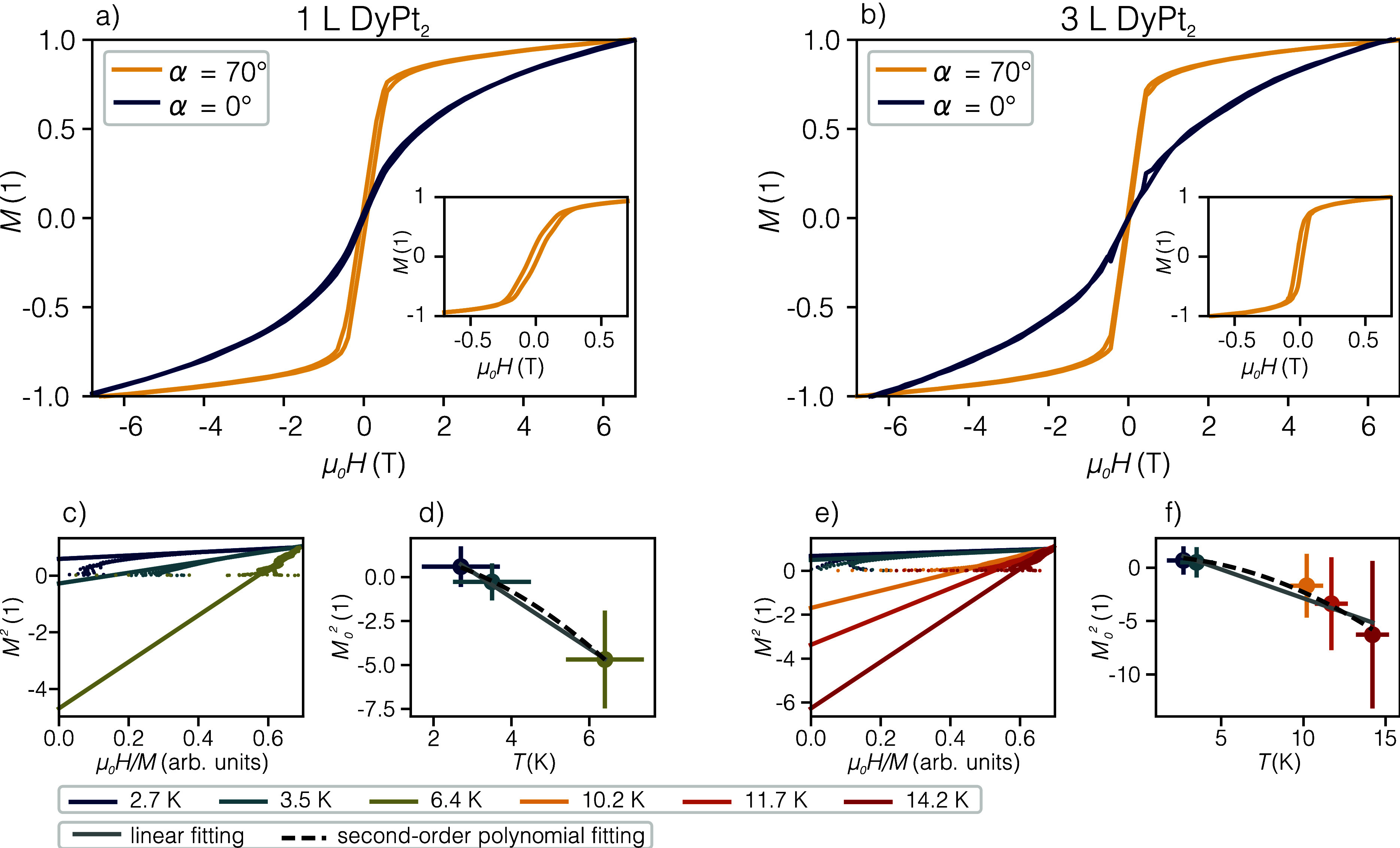
XAS/XMCD results taken at 3 K unless otherwise stated. Left panel shows the results for 1 L DyPt_2_, while the right for 3 L DyPt_2_. (a and b) XMCD hysteresis loops for normal (blue) and grazing (yellow) light incidence configurations at the energy of M_5_ absorption edge for the magnetic field range ±6.8 T. Insets: XMCD hysteresis loops for grazing incidence in magnetic field range ±0.7 T to show the loop opening. (c and e) Arrott plots derived from the in-plane magnetization loops at various temperatures. (d and f) Dependence of *M*_0_^2^ intercept as a function of temperature and two fitting methods used to estimate the *T*_C_.

*T*_C_ is determined using the Arrott plot method by recording the magnetization loops for various temperatures^42^ as shown in Figure 5c,e. The linear fitting of high magnetic field data intersects the *y*-axis at different *M*_0_^2^ points. Figure 5d,f shows the dependence of the intercept as a function of temperature. Two types of trend lines were fitted to the data: the second-order polynomial function^6,8,10^ and linear fitting.^32^ From linear fitting the *T*_C_ are 3 ± 1 and 5 ± 1 K for 1 L DyPt_2_ and 3 L DyPt_2_, respectively. Fitting of the quadratic trend line gives the same value of *T*_C_ for 1 L DyPt_2_ and an overestimated value of *T*_C_ equal to 6 ± 1 K for 3 L DyPt_2_. It indicates that the base temperature of measurements is already close to the Curie point for both thicknesses of surface alloy, thus the linear fitting seems more likely. Uncertainty factors of *M*_0_^2^ result from the goodness of fitting of a linear function to the *M* ^2^(μ_0_ *H*/*M*). The *T*_C_ derived for 1 L DyPt_2_ and 3 L DyPt_2_ are significantly lower compared to the bulk DyPt_2_ alloy (25–29 K).^41,43^ Contrary to cases of GdAu_2_ and GdAg_2_,^8^ DyPt_2_ shows weakening of magnetic ordering resulting in a smaller *T*_C_. It is in line with the possible noncollinear component of the magnetization between planes, reducing the ordering and leading to 3 L DyPt_2_ having a smaller *T*_C_ than 1 L DyPt_2_.

## Conclusions

In summary, we determined the growth conditions of a single layer of DyPt_2_ surface alloy as well as its triple layer using the reactive growth of Dy on a Pt(111) single crystal. The atomic structure of termination layer of both thicknesses of surface alloy is much the same with a slightly bigger unit cell of 3 L DyPt_2_. Both thicknesses differ however with the moiré pattern that is more ordered for triple layer. Electronic properties of both are similar and they are a combination of Dy and Pt 5d and 6s states, without the dominance of any. Alloying reduces the work function of the pure substrate and its value is dependent on the coupling strength between the surface alloy and substrate, which is in line with the moiré pattern modulation. Independently of the number of layers, DyPt_2_ surface alloys are soft ferromagnetic materials with low *T*_C_ values on the order of a few Kelvins and an in-plane easy magnetization axis. Experimentally estimated orbital and spin magnetic moments are lower than those for the Dy^3+^ ion, indicating the charge transfer and polarization of Pt atoms in close proximity to Dy. Owing to the 1 L DyPt_2_ and 3 L DyPt_2_ surface alloy moiré pattern, and since materials with pronounced moiré are known to induce templated growth of clusters and/or molecules,^23,44,45^ in combination with their low *T*_C_, these systems are promising substrates to investigate steered templated magnetic structures.

## Supplementary Material



## Notes

Analysis of growth condition influence on the sample area occupancy by Dy–Pt surface alloy and other structures, detailed temperature-dependent studies of the surface morphology, LEED results, electronic properties of 3 L DyPt_2_ dependence on moiré pattern, energetic position dependence on moiré pattern analysis, normal geometry XAS, and experimental section (PDF)
