# Supplementary material for: Ferromagnetism in Two-Dimensional Dysprosium–Platinum Surface Alloy
Source: Nano Lett. 2025 May 17;25(24):9519–25. doi: 10.1021/acs.nanolett.5c00262 (PMC12186622; doi:10.1021/acs.nanolett.5c00262)
Supplement: Supplementary file 1 [file nl5c00262_si_001.pdf]

Supporting Information for Ferromagnetism in Two-Dimensional Dysprosium-Platinum Surface Alloy

Marta Przychodnia*,† and Maciej Bazarnik‡

†*Institute of Physics, Poznan University of Technology, 3 Piotrowo Street, 60-965 Poznan, Poland*

‡*Physikalisches Institut, Münster University, Wilhelm-Klemm-Str. 10, 48149 Münster, Germany*

E-mail: marta.przychodnia@put.poznan.pl

Supplementary note 1:

Analysis of growth conditions influence on sample area occupancy by Dy-Pt surface alloy and other structures.

A variety of samples were analyzed to understand how the surface structures are affected by the growth parameters such as sample temperature, Dy coverage during reactive growth, and post-annealing time. The results showed that the latter has no impact on the sample morphology and only two factors determine the contribution to surface area occupancy: the initial surface coverage with Dy and substrate temperature during the reactive growth and post-annealing processes. The temperature dependence is described in detail in Supplementary Note 2, but in general, it is necessary to keep the temperature in the range above the alloy formation activation temperature and below its overheating temperature. STM is

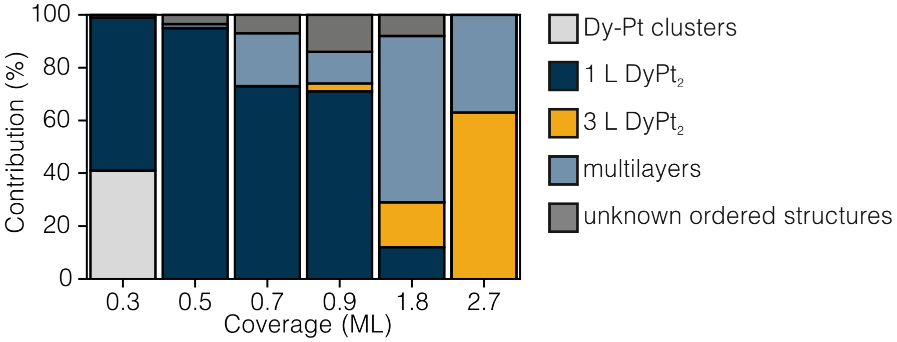

Figure S1. Diagram presents the contribution of Dy-Pt surface alloy and other structures in the sample area occupation as a function of Dy coverage. All samples used for this investigation were prepared using the same procedure of a reactive growth process with a substrate temperature of 1050 K and various Dy coverage in a range from 0.3 ML to 2.7 ML.

a local technique thus to collect reliable statistics, the macroscopic position of the sample was changed at least three times and numerous large-scale STM topography images were collected with a total area of at least 1.5 μm^2. To preserve the most constant level of Dy diffusion into the bulk for coverage-dependent analysis, only the deposition rate was increased while the deposition time was kept. Coverage dependence of Dy-Pt surface alloy and other structures contribution into sample surface occupancy is summarized in Figure S1. For Dy coverage less than 0.4 ML, the surface is primarily occupied in 41 % with Dy-Pt clusters with unknown stoichiometry and in 58 % with 1 L $DyPt_2$. Local multilayer areas cover the remaining 1 % of the surface. At 0.5 ML Dy coverage, almost the entire sample area is occupied by 1 L $DyPt_2$ (95 %). As Dy coverage increases to 0.7 ML and 0.9 ML, the sample is still dominated by 1 L $DyPt_2$ which covers 70-75 % of the sample. The remaining sample's surface is covered by multilayers and other unknown ordered structures, that were not deeply investigated because of their small share in the surface coverage. In the case of the 0.9 ML coverage small areas (3 %) covered with 3 L $DyPt_2$ appear. This behavior indicates a Stranski–Krastanov growth mode. The surface composition observed for the Dy coverage of 1.8 ML, where the sample is dominated by multilayers, is unclear. This domination may result from local structure inhomogeneity. Most likely, the areas with locally increased density of multilayers were imaged here. When Dy coverage reaches 2.7 ML, the

surface is dominated by 3 L $DyPt_2$, and the remaining part (about 37 %) is occupied by multilayers.

Supplementary note 2:

Detailed temperature-dependent studies of the surface morphology.

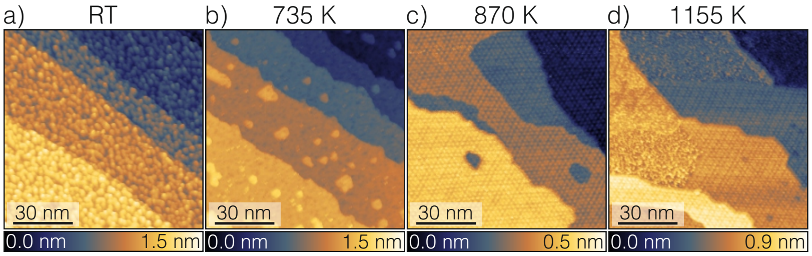

Figure S2. STM topography images showing thermal evolution of Dy-Pt surface structures – a) 0.3 ML deposition on substrate with a RT; b) 0.3 ML deposition on substrate with a temperature of 735 K; c) 0.4 ML deposition on substrate with a temperature of 870 K; d) 0.4 ML deposition on substrate with a temperature of 1155 K. Tunneling parameters: a) $I_t = 1.2$ nA, $U = 1.5$ V, b) $I_t = 0.6$ nA, $U = 1.5$ V, c) $I_t = 1.2$ nA, $U = 1.5$ V, d) $I_t = 1.7$ nA, $U = 1.3$ V.

When Dy is deposited onto a clean Pt(111) substrate at RT, it does not create ordered structures, just as previously observed in the Ce-Pt [1,2] and Gd-Pt[3] systems. Instead, Dy is forming irregular-shaped and randomly distributed clusters and islands with $(150 - 230) \pm 5$ pm apparent height (Figure S2 a)). When reactive growth occurs below the alloy-formation temperature, Dy and Pt atoms intermix and form irregularly shaped islands with unknown stoichiometry (Figure S2 b)). Only when the alloy-formation activation temperature is reached, regardless of annealing time, the ordered structures of the surface alloy are formed (Figure S2 c)). The Dy-Pt system's alloy-formation activation temperature is approximately 780 ± 5 K, which is very close to the activation temperature reported for the Gd-Pt system.[3,4] It is known that Pt tends to form overlayers not only due to the electrochemical reactions[5] but also due to the annealing at the high temperature.[4,6] When the temperature of the Dy-Pt system exceeds 1165 ± 5 K, samples get overheated, and Pt overlayers form (see Figure S2 d)). The temperature window for surface alloy growth is limited by the activation energy on the

lower range and the formation of Pt overlayers on the upper range.

Detailed analysis of surface structures observed within the alloy formation temperature range reveals that the Dy-Pt system does not form $DyPt_5$ surface alloy as it is observed for Gd-Pt,[3,4] Ce-Pt,[7,8] La-Pt[9,10] systems. $REM-Pt_5$ surface alloy is formed by the thermal evolution of the $REM-Pt_2$ alloy into $REM-Pt_5$, as a result of providing sufficiently high energy, to form Pt overlayer on top of $REM-Pt_2$. In the case of the Dy-Pt system, we do not observe surface alloy transformation with the temperature. The samples covered with nearly 0.4 ML Dy and prepared with various temperatures show only small fluctuations (of the order of measurement uncertainty) of the area occupied by 1 L $DyPt_2$ and other minority structures.

Supplementary Note 3:

Low-energy electron diffraction analysis.

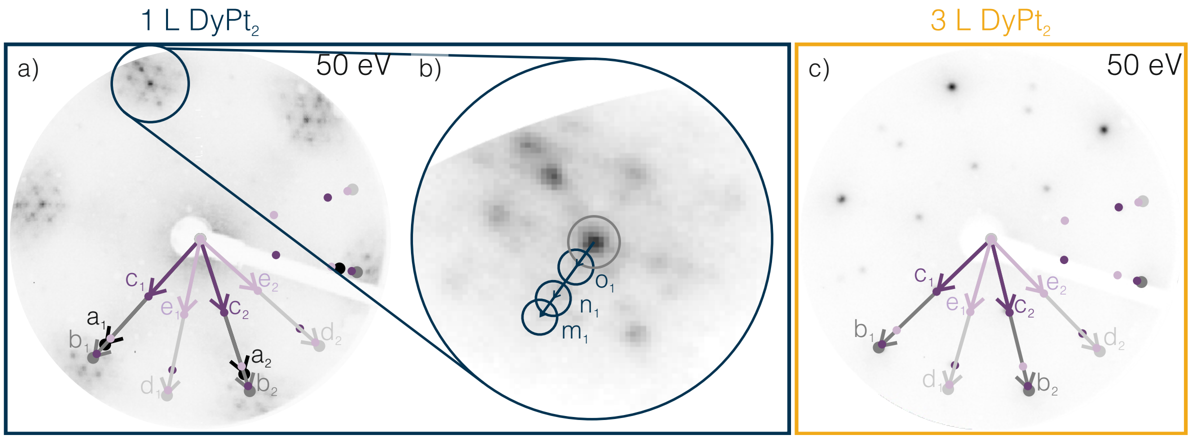

Figure S3. Experimental LEED diffraction images taken for electrons energy of 50 eV with simulated LEED patterns overlaid on the half of image of a) 1 L $DyPt_2$, b) zoom-in on the satellites spots, and c) 3 L $DyPt_2$. Black vectors $(\mathbf{a_1}, \mathbf{a_2})$ points Pt(111) structure. Gray vectors ($\mathbf{b_1}, \mathbf{b_2}$ and $\mathbf{d_1}, \mathbf{d_2}$) point the lattice of Pt atoms forming surface alloy, not rotated and rotated relative to the substrate, respectively. Purple vectors ($\mathbf{c_1}, \mathbf{c_2}$ and $\mathbf{e_1}, \mathbf{e_2}$) indicate the lattice formed by Dy atoms from alloy layers that are not rotated and rotated relative to the substrate, respectively. Blue vectors $\mathbf{m_1}, \mathbf{n_1}, \mathbf{o_1}$ indicates moiré pattern periodicities. Single vectors are indicated to keep the image transparent.

Figure S3 shows the results of the LEED experiment combined with simulated structure

in the reciprocal lattice of 1 L $DyPt_2$ and 3 L $DyPt_2$. The Pt(111) substrate signal (marked with $\mathbf{a_1}, \mathbf{a_2}$ vectors) is visible only for 1 L $DyPt_2$ (Figure S3 a)). The diffraction spots are among overlaying satellites around the main diffraction spots of the nearest neighbor distance of surface alloy. Vectors $\mathbf{b_1}, \mathbf{b_2}$ and $\mathbf{d_1}, \mathbf{d_2}$, indicating the interatomic distance of nearest atoms in surface alloy, form (0.8881×0.8881) and $(0.8881 \times 0.8881)R30°$ superstructures in relation to the Pt(111) substrate. There are two rotational domains, one parallel ($\pm 3°$) to the substrate and the other rotated by $30 \pm 3°$. Both reveal lattice constant contraction of about 11 % against the pure Pt(111) lattice constant, which is possible due to the structure buckling, already observed for $GdPt_2$ surface alloy.[3] Assuming that the same is the case for Dy-Pt, the Dy atoms are pushed out from the alloy layer, and the Pt atoms surrounding Dy may form a kagomé-like lattice around them with a contracted lattice constant. Due to the structure buckling another superstructure formed by Dy atoms, marked with vectors $\mathbf{c_1}, \mathbf{c_2}$ and $\mathbf{e_1}, \mathbf{e_2}$ is observed. Following the Pt, Dy lattices occur in two rotational domains (1.8375×1.8375) and $(1.8375 \times 1.8375)R30°$ in relation to the Pt(111) substrate. Satellites, described by $\mathbf{m_1}, \mathbf{n_1}, \mathbf{o_1}$ vectors in zoom-in of Figure S3 b), arise from slight differences in interatomic distances resulting from the moiré pattern.[11] Numerous satellite spots confirm the complex and highly disturbed structure of the moiré pattern. Three main vectors whose lengths point to the moiré pattern constants of 3.6 ± 0.2 nm, 5.0 ± 0.2 nm, and 8.8 ± 0.2 nm are observed when enlarged. They correspond respectively to contracted (2×2), relaxed (2×2), and (4×4) of apparent moiré pattern's unit cells, revealed in STM topography shown in the Figure 1 e) of the manuscript. There is no signal from the Pt(111) substrate observed for 3 L $DyPt_2$ (Figure S3 c)). Vectors $\mathbf{b_1}, \mathbf{b_2}$ and $\mathbf{d_1}, \mathbf{d_2}$ form (0.9025×0.9025) and $(0.9025 \times 0.9025)R30°$ superstructures with regard to the Pt(111) substrate. The Pt lattice is less contracted than in the case of 1 L $DyPt_2$ most probably due to the structure relaxation with increased layers number. Superstructures formed by vectors $\mathbf{c_1}, \mathbf{c_2}$ and $\mathbf{e_1}, \mathbf{e_2}$ are (1.8592×1.8592) and $(1.8592 \times 1.8592)R30°$ with respect to the Pt(111) substrate. The shadows around the main atomic nearest neighbor distance spots are the only fingerprints

of the moiré pattern pointing to the very large unit cell and/or non-uniform moiré pattern. The presented LEED images were selected as representatives of a wide energy range of data, and both were taken for electron energy 50 eV. Any differences between the reciprocal and real space dimensions, especially the species on the LEED images' edges, may be caused by distortions due to the screen curvature.

Supplementary Note 4:

Electronic properties of 3 L $DyPt_2$ dependence on moiré pattern.

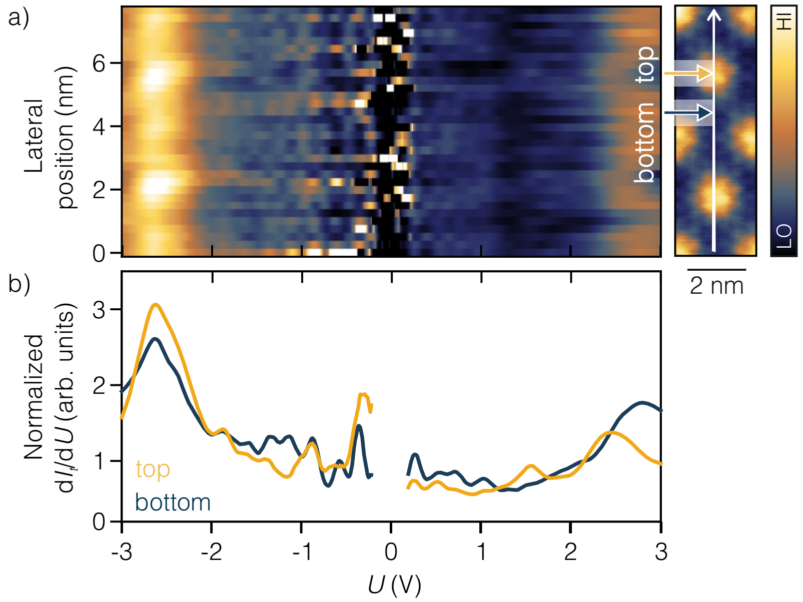

Figure S4. Moiré pattern influence on electronic properties of 3 L $DyPt_2$. a) Waterfall plot of 32 normalized CC STS spectra taken along the white arrow marked in STM topography on the right side. The noise around 0 V bias results from the normalization procedure. b) Two curves selected from the dataset shown in a) for the two extreme positions of the peak around 2.9 V corresponding to the top (yellow) and the bottom (blue) positions on the moiré pattern. The spectra' positions are also marked in the topography with arrows. The energy range around 0 V bias was omitted due to the normalization procedure noise. Tunneling parameters: $I_{stab} = 5$ nA, $U_{stab} = 3$ V, $U_{mod} = 50$ mV, $\omega_{mod} = 49.95$ kHz.

The moiré dependence of the electronic structure of 3 L $DyPt_2$ is analyzed in the same manner as presented in the manuscript for 1 L $DyPt_2$. The white arrow in STM topography points to the line across which the 32 CC STS were recorded. As for 1 L $DyPt_2$ waterfall plot

(Figure S4 a)) exposes signal intensity modulation that is caused by the varying coupling strength of the surface alloy with the substrate. Based on the energetic position of the maximum of unoccupied states two extreme curves are separately presented in Figure S4 b). The peak shifted toward low energies is observed for the top site, while the peak shifted toward high energies for the bottom site of the moiré unit cell, following the observation for 1 L $DyPt_2$. One pronounced maximum, attributed to Pt d states[12] is observed on the occupied side at -2.70 ± 0.05 V. Its energetic position seems to be constant within error across the moiré unit cell, while its intensity follows the structure modulation reaching the maximum for the top sites of moiré pattern. The lower-intensity states are the combination of 5d and 6s states of Pt, with the domination of the former one. Maximum of unoccupied states, corresponding to the 5d states of Dy, also exposes two merged peaks with the same positions within the error (2.55 ± 0.05 V and 2.90 ± 0.05 V) as for 1 L $DyPt_2$. The difference is that in the case of 3 L $DyPt_2$, the latter one has a higher intensity. The remaining states on the unoccupied side are the combination of 5d and 6s states of Pt and REM.

Supplementary Note 5:

Energetic position dependence on moiré pattern analysis.

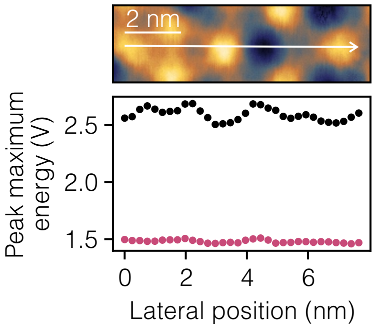

Figure S5. Graph of energetic positions of two unoccupied states peaks as a function of lateral position within the moiré pattern.

Figure S5 presents the energetic position dependence of the two most pronounced unoccupied peaks of 1 L $DyPt_2$ on lateral position within moiré unit cell. The maxima positions

were extracted using Gaussian fitting. The higher-energy peak shows the dependence on moiré modulation while the peak at 1.5 V seems to be independent. The highest value is observed for the bottom sites of the moiré pattern while the top sites reach the lowest value. Based on this analysis, two extreme curves were selected from the 32 spectra dataset and presented separately for both thicknesses of surface alloy (manuscript Figure 2, and SI Figure S4).

Supplementary Note 6:

Normal geometry XAS spectra.

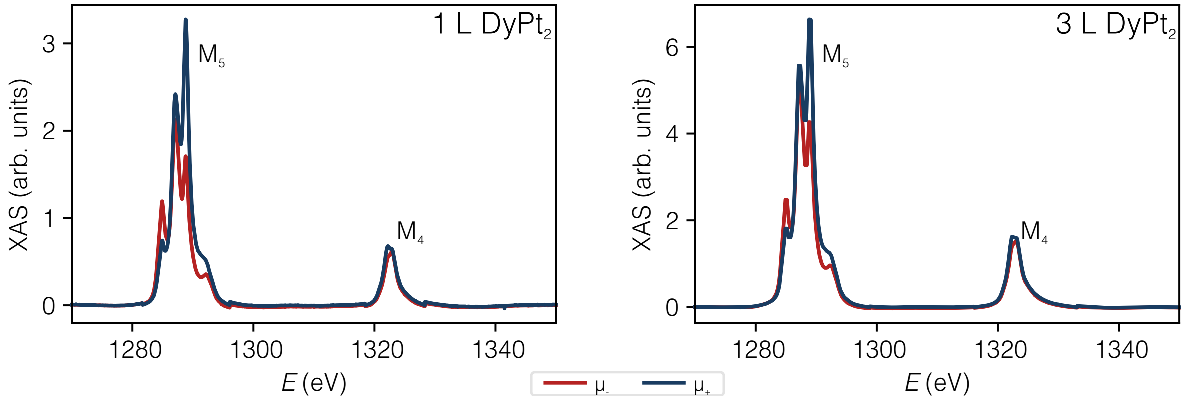

Figure S6. Normal geometry XAS spectra for positive (blue) and negative (red) light polarization for 1 L $DyPt_2$ and 3 L $DyPt_2$. Measurement taken at 3 K and 6.8 T.

XAS spectra for out-of-plane geometry (normal ($\alpha = 0°$) beam–sample configuration) with an external magnetic field of $\mu_0 H = 6.8$ T applied along the beam direction recorded using the left (red line) and right (blue line) circularly polarized light. The difference between positive and negative polarization light absorption is visible in both cases pointing to the dichroism of two absorption channels, namely M_5 and M_4 absorption edges. Presented XAS spectra were used to extract the XMCD signal presented in the Figures 4 a) – b) of the manuscript.

Experimental

Samples preparation

Samples were prepared *in situ* in ultra-high vacuum (UHV) systems where the experiments were performed. The STM, STS, and LEED experiments were performed in a multi-chamber UHV system with a base pressure of 5.0×10^{-11} mbar. XAS/XMCD experiments were performed at the X-Treme beamline at the Swiss Light Source of Paul Scherrer Institute.

The Pt(111) single-crystal substrate with a purity of 99.999%, was supplied by MaTecK, GmbH, Germany. It was cleaned by repeated cycles of Ar-ion bombardment, heating in an oxygen atmosphere ($p_{O_2} = 5.0 \times 10^{-8}$ mbar), and flashing up to 1300 K. This procedure was repeated until the number of impurities on the surface was less than 2 %. Before Dy deposition, the substrate was characterized using STM imaging, STS, and LEED techniques to verify surface cleanliness. The surface alloy was grown using a reactive growth process, where Dy was evaporated from a tungsten crucible, using an e-beam evaporator, onto the substrate held at temperatures ranging from room temperature (RT) to 1165 K. During the deposition processes, the background pressure did not exceed 1.0×10^{-10} mbar. To preserve the same level of Dy diffusion into the bulk for coverage-dependence analysis, we increased only the deposition rate, while the deposition time was kept constant. The coverage (θ) of one monolayer ML is assigned to the amount of deposited Dy necessary to form a densely-packed single-atom-thick layer of Dy atoms yielding 9.1 Dy atoms per nm^2. After deposition, the sample was kept at the deposition temperature for 5 minutes. To ensure reproducible deposition conditions, we controlled the substrate temperature in two ways: by calibration of the heating stage based on a thermocouple readout, and by an externally mounted pyrometer focused on the sample. Afterward, the sample was transferred *in vacuo* into the analysis chamber and cooled to the measurement temperature.

Scanning tunneling microscopy and spectroscopy

STM and STS experiments were performed using a home-built variable-temperature STM head operating at 70 K. Electrochemically etched tungsten probes cleaned by standard *in situ* procedures were used for the STM studies. All topographic data were obtained in CC mode and processed using GWYDDION software.[13] STS in the range from -3 V to 3 V were recorded in CH mode after probe stabilization at given parameters, *i.e.* bias (U) and tunneling current (I_{t}). The differential tunneling conductance ($\mathrm{d}I_{\mathrm{t}}/\mathrm{d}U$) was measured via lock-in technique by applying modulation voltage to the sample bias voltage with a peak-to-peak value $U_{\mathrm{pk-pk}} = 50$ mV and a frequency $\nu = 6.8$ kHz. CH $\mathrm{d}I_{\mathrm{t}}/\mathrm{d}U$ spectra were normalized over I_{t}/U leading to artifacts around zero bias. However, at the measurement temperature of 70 K, we do not observe any features around Fermi energy in the raw $\mathrm{d}I_{\mathrm{t}}/\mathrm{d}U$ data, thus the region from -0.01 V to 0.01 V in the normalized data has been omitted. Resonant STS was performed using CC mode by positioning the probe at a measurement point and ramping U in the range from 1 V to 10 V while recording the $z(U)$ and $\mathrm{d}I_{\mathrm{t}}/\mathrm{d}U$ characteristics. The $\mathrm{d}I_{\mathrm{t}}/\mathrm{d}U$ was measured via lock-in technique by applying modulation voltage to the sample bias voltage with a peak-to-peak value $U_{\mathrm{pk-pk}} = 50$ mV and a frequency $\nu = 49.95$ kHz. IPSs energies were extracted from each $\mathrm{d}I_{\mathrm{t}}/\mathrm{d}U$ curve using the Gaussian function fitting. Further, a simple model of Schrödinger equation's solution for the triangular potential barrier, described in detail in,[14] is implemented to calculate the local surface work function Φ:

$$E_n = \frac{\Phi}{e} + \alpha\left(n - \frac{1}{4}\right)^{\frac{2}{3}} F^{\frac{2}{3}},$$

where E_n is the energy of the n-th IPS, e is the elementary charge, $\alpha = \left(\frac{\hbar^2}{2me}\right)^{\frac{1}{3}}\left(\frac{3\pi}{2}\right)^{\frac{2}{3}}$ is the constant, F is the electric field. Plotting the E_n as a function of $\left(n - \frac{1}{4}\right)^{\frac{2}{3}}$ results in a straight line, whose intercept with y-axis provides the surface work function value. IPS's order is assigned following the procedure described by Schlenhoff.[14] All spectroscopic data were processed using self-written Python scripts.

Low-energy electron diffraction

LEED experiments were performed directly after STM/STS characterization in the same analysis chamber using a commercial LEED/AES spectrometer from OCI Vacuum Microengineering Inc. The LEED images presented in Supporting Information are shown for E_{beam} = 50 eV, and they are only a representative example of the full range of data collected for an electron beam energy from 20 eV to 250 eV in 5 eV steps. LEED patterns were analyzed and simulated using LEEDLab software.[15,16] LEED images were calibrated using a pristine Pt(111) LEED pattern. Simulated LEED patterns are reproduced using dimensions extracted from STM and they are overlaid on half of the experimental LEED data.

X-ray magnetic circular dichroism

XAS/XMCD experiments were performed at the X-Treme beamline of the Swiss Light Source, Paul Scherrer Institute, Villigen, Switzerland. Samples were prepared *in situ* according to the procedure described above, and verified via LEED and STM prior to the experiment. During the measurements, the sample was kept at 3 K, following the beamline temperature calibration. The only exceptions were temperature-dependent magnetization loops used for the calculation of T_{C}, where the temperature is indicated in the description. XAS were recorded in the photon beam energy range covering Dy $M_{4,5}$ edges for the sample magnetized with a magnetic field $\mu_0 H = 6.8$ T. Before the calculation of the XMCD signal, the XAS spectra were normalized over the ring current, registered during the measurement. Although the background step signal registered in the spectra is small compared to the XAS intensity, the Shirley function was calculated and subtracted from each XAS spectrum to reduce the error input to the sum rules calculations. Finally, to compare results for various samples, the XMCD signal was normalized over the maximum averaged XAS signal for a given sample. Spin and orbital magnetic moments were extracted using sum rules following Thole[17] and Carra.[18] XMCD hysteresis loops were recorded for two photons' energies – one that matches with the maximum of the XMCD signal of the M_5 absorption edge and

the other, which corresponds to the energy prior to the absorption edge (called pre-edge) for normalization purposes. The signals for both energies were recorded alternately while the magnetic field was successively sweeping from −6.8 T to 6.8 T and back. Such loops were measured for both light polarizations. For normalization purposes, the edge signal is divided over the pre-edge signal, and then the signals for both polarizations are subtracted and divided over their sum ($\frac{C^- - C^+}{C^- + C^+}$). Finally, to compare results for various samples, the hysteresis loops were normalized over the signal at the highest magnetic field for a given sample. All of the measurements were carried out for two beam – sample configurations (normal $\alpha = 0$ and grazing $\alpha = 70$) giving *out-of-plane* and *in-plane* contrast to determine the easy magnetization axis.

References

(1) Baddeley, C. J.; Stephenson, A. W.; Hardacre, C.; Tikhov, M.; Lambert, R. M. Structural and electronic properties of Ce overlayers and low-dimensional Pt-Ce alloys on Pt111. *Physical Review B - Condensed Matter and Materials Physics* **1997**, *56*, 12589–12598.

(2) Tang, J.; Lawrence, J. M.; Hemminger, J. C. Structure and valence of the Ce/Pt(111) system. *Physical Review B - Condensed Matter and Materials Physics* **1993**, *48*, 15342–15352.

(3) Przychodnia, M.; Hermanowicz, M.; Sierda, E.; Elsebach, M.; Grzela, T.; Wiesendanger, R.; Bazarnik, M. Controlled growth of Gd-Pt surface alloys on Pt (111). *Physical Review B - Condensed Matter and Materials Physics* **2022**, *105*, 035416.

(4) Ulrikkeholm, E. T.; Pedersen, A. F.; Vej-Hansen, U. G.; Escudero-Escribano, M.; Stephens, I. E.; Friebel, D.; Mehta, A.; Schiøtz, J.; Feidenhansl', R. K.; Nilsson, A.;

Chorkendorff, I. PtxGd alloy formation on Pt(111): Preparation and structural characterization. *Surface Science* **2016**, *652*, 114–122.

(5) Pedersen, A. F.; Ulrikkeholm, E. T.; Escudero-Escribano, M.; Johansson, T. P.; Malacrida, P.; Pedersen, C. M.; Hansen, M. H.; Jensen, K. D.; Rossmeisl, J.; Friebel, D.; Nilsson, A.; Chorkendorff, I.; Stephens, I. E. Probing the nanoscale structure of the catalytically active overlayer on Pt alloys with rare earths. *Nano Energy* **2016**, *29*, 249–260.

(6) Stephens, I. E.; Bondarenko, A. S.; Grønbjerg, U.; Rossmeisl, J.; Chorkendorff, I. Understanding the electrocatalysis of oxygen reduction on platinum and its alloys. *Energy and Environmental Science* **2012**, *5*, 6744–6762.

(7) Essen, J. M.; Becker, C.; Wandelt, K. PtxCe1-x Surface Alloys on Pt(111): Structure and Adsorption. *e-Journal of Surface Science and Nanotechnology* **2009**, *7*, 421–428.

(8) Klein, M.; Nuber, A.; Schwab, H.; Albers, C.; Tobita, N.; Higashiguchi, M.; Jiang, J.; Fukuda, S.; Tanaka, K.; Shimada, K.; Mulazzi, M.; Assaad, F. F.; Reinert, F. Coherent heavy quasiparticles in a CePt5 surface alloy. *Physical Review Letters* **2011**, *106*, 4–7.

(9) Malacrida, P.; Escudero-Escribano, M.; Verdaguer-Casadevall, A.; Stephens, I. E. L.; Chorkendorff, I. Enhanced activity and stability of Pt–La and Pt–Ce alloys for oxygen electroreduction: the elucidation of the active surface phase. *Journal of Materials Chemistry A* **2014**, *2*, 4234–4243.

(10) Tereshchuk, P.; Piotrowski, M. J.; Da Silva, J. L. Atomic structure of the La/Pt(111) and Ce/Pt(111) surfaces revealed by DFT+U calculations. *RSC Advances* **2015**, *5*, 521–528.

(11) Mousavion, S.; Yu, K. M.; Maniraj, M.; Lyu, L.; Knippertz, J.; Stadtmüller, B.; Aeschlimann, M. Atomic and mesoscopic structure of Dy-based surface alloys on noble metals. *New Journal of Physics* **2022**, *24*, 033048.

(12) Smith, N. V.; Wertheim, G. K.; Hufner, S.; Traum, M. M. Photoemission spectra and band structures of d-band metals. *Physical Review B - Condensed Matter and Materials Physics* **1974**, *10*, 3197–3206.

(13) Nečas, D.; Klapetek, P. Gwyddion: An Open-Source Software for SPM Data Analysis. *Cent. Eur. J. Phys.* **2012**, *10*, 181–188.

(14) Schlenhoff, A.; Kokkorakis, G. C.; Xanthakis, J. P. High-Order Gundlach Resonances at Exceptional Large Voltages: Consequences for Determining Work Functions. *Appl. Phys. Lett.* **2022**, *120*, 261601.

(15) F. Sojka and T. Fritz, Fritz & Sojka GbR, Apolda Germany, 2019 LEEDLab 2018. `https://fritz-sojka-gbr.de/leedlab/`.

(16) Sojka, F.; Meissner, M.; Zwick, C.; Forker, R.; Fritz, T. Determination and Correction of Distortions and Systematic Errors in Low-Energy Electron Diffraction. *Rev. Sci. Instrum.* **2013**, *84*, 015111.

(17) Thole, B. T.; Carra, P.; Sette, F.; Van Der Laan, G. X-ray Circular Dichroism as a Probe of Orbital Magnetization. *Phys. Rev. Lett.* **1992**, *68*, 1943–1946.

(18) Carra, P.; Thole, B. T.; Altarelli, M.; Wang, X. X-ray Circular Dichroism and Local Magnetic Fields. *Phys. Rev. Lett.* **1993**, *70*, 694–697.
